# A Correction

**Published:** 1846-05

**Authors:** 


### A CORRECTION.

We take great pleasure in complying with the request of the publishers of Ellis's Medical Formulary, Messrs. Lea & Blanchard, to insert the following correction of a serious error in that work, and beg leave to call the attention of our readers to it.

"*Ellis's Medical Formulary.—Correction.*—The publishers of this work respectfully request those persons who have the seventh edition, to correct a typographical error for the "*Medicated Hydrocyanate of Potassium,*" at page 83; wherein the symbol for an ounce is used in place of that for a drachm. The following is the correct prescription, and corresponds with the proportions directed in all the previous editions of the work:

℞ Potassii hydrocyanici medicati, ʒj.
Aquæ destillatæ, Oj.
Sacchari purificati, ℥iss.

Fiat solutio.—Dose, a table-spoonful, night and morning.

